# Pancytopenia in a surgical patient, a rare presentation of hyperthyroidism

**DOI:** 10.1186/1471-2482-14-108

**Published:** 2014-12-15

**Authors:** Prabhat Jha, Yogendra Prasad Singh, Bikal Ghimire, Binit Kumar Jha

**Affiliations:** Department of Surgery, Institute of Medicine, Tribhuvan University Teaching Hospital, Kathmandu, Nepal

**Keywords:** Hyperthyroidism, Pancytopenia

## Abstract

**Background:**

Pancytopenia is a rare complication of hyperthyroidism. Various mechanisms have been described such as immunological, bone marrow suppression. The possibility of hyperthyroidism should be considered in patients with unexplained pancytopenia. There are many case reports showing the association between hyperthyroidism and pancytopenia. All of these reports show association between Graves disease and pancytopenia but our case shows association between Multinodular goitre and pancytopenia. Besides it is uncommon to find such association in a surgical patient.

**Case presentation:**

This case report describes a 62 yr old hindu female with splenic injury and pancytopenia. On further investigations the patient was found to have hyperthyroidism.

**Conclusion:**

Though the definite mechanism regarding the association of pancytopenia with hyperthyroidism isn’t clear, various cases have been described in the literature. This case shows the diagnostic dilemma that can occur in patients with pancytopenia. Any patient with unexplained pancytopenia should undergo thyroid function tests to rule out hyperthyroidism.

## Background

Atypical manifestations of hyperthyroidism include hematological, cardiovascular, dermatological. Hyperthyroidism can be associated with various hematological disorders. Single lineage abnormalities such as anemia (34%), leukopenia (5.8%), thrombocytopenia (3.3%) have been reported, but pancytopenia is a rare presentation [1–4]. The suspected etiologic mechanisms include ineffective hematopoiesis, reduction in blood cell life span, autoimmune process [5, 6], toxicity of thyroid hormone.

Cases have been described which show an association between graves’ disease and pancytopenia but no case reports have described association between multinodular goitre and pancytopenia. Also this is the first case report describing association between hyperthyroidism and pancytopenia in a surgical patient.

## Case presentation

A 62 year old female presented to the emergency room with history of fall from one storey building. The impact was over the trunk region. On clinical examination the blood pressure was 90/60 mmHg, pulse rate was 110/min, temperature was 98.6 degree Farenheit and respiratory rate was 20/min. Pallor was present and the thyroid gland was enlarged. Pansystolic murmur was present over the mitral and tricuspid regions. Fine basal crepitations were heard over both lung fields. Left upper quadrant tenderness was present.

A complete blood count showed pancytopenia with hemoglobin 7.4gm/dl (normal: 12–16 gm/dl), WBC count 3400/μl (normal: 4000–11,000/μl) and platelet count 91,000/μl (normal 1,50,000-4,00,000/μl). Multiple ventricular premature complexes were present in the electrocardiogram (ECG). Ultrasound of the abdomen showed splenic laceration with splenic hematoma extending up to the hilum. Contrast enhanced computed tomography of the abdomen showed findings consistent with grade II splenic injury.

The patient was admitted to the intensive care unit (ICU) for conservative management and further workup with the impression of grade II splenic injury and pancytopenia.

Ultrasound of the neck showed multiple heteroechoic nodules with calcification in both lobes of thyroid, findings suggestive of multinodular goiter. Echocardiography showed severe tricuspid regurgitation and mitral regurgitation. Bone marrow aspiration showed mixed normocellular and a few hypercellular marrow fragments. Absolute retics and peripheral blood smear were normal. Hormonal studies showed features suggestive of hyperthyroidism (Table 1).

**Table 1:** **Thyroid function test at admission**

Investigation	Values at admission	Reference range
T3	7.4	4.2-8.1 pmol/l
T4	35	10-28.2 pmol/l
TSH	<0.015	0.4-4.6 pmol/l

The patients hemoglobin improved with two units of packed cells transfusion but the WBC and platelet count didn’t improve. On the third day of admission, she developed a temperature of 102 degree Farenheit, pulse rate of 150-160/min, respiratory rate of 30/min and oxygen saturation of 75-80% at 5 L/min of oxygen via face mask. On examination the patient was agitated and had diffuse crepitations over both lung fields. On ECG monitoring multiple VPCs per minute was present. A Burch Wartofsky Score of 95 was calculated (Table 2). With the impression of thyroid storm, the patient was started on hydrocortisone, oral propranolol 40 mg q6hrly (intravenous preparation not available), and oral carbimazole 10 mg q8hrly. After 24 hours the patients pulse rate stabilized between 80–100 bpm, temperature was 99 degree farenheit and there were only few VPCs per minute. Propranolol was gradually tapered over a period of one week and her pulse rate stabilized at 60 to 80 bpm. After starting carbimazole, her WBC and platelet counts started improving and became normal at discharge (Table 3).

**Table 2:** **Burch Wartofsky score calculation**

Clinical and physical criteria	Point
Temperature 102–102.9 degree F	20
Central Nervous Effect (Mild Agitation)	10
Hepatogastrointestinal dysfunction (nausea, vomiting)	10
Cardiovascular Dysfunction Pulse rate > =140/min	25
Moderate Bibasilar Rales	10
Arrythmia Present	10
Suggestive History Present	10
**Total points**	**95**

**Table 3:** **Investigations at admission and improvement of pancytopenia with treatment**

Hematologic parameters	Day of treatment					
	1	2	3	4	5	8
Hemoglobin (gm %)	7.4	9	9.6	9.6	11.3	11.6
PCV %	22.9	26.7	29.2	29.2	35.1	35.7
Total leukocyte count (/mm^3^)	4000	3400	3100	3000	3100	3900
Platelets (/cumm)	91000	89000	128000	112000	112000	104000

The patient was advised for radionuclide thyroid scan and further treatment.

## Discussion

The patient had multinodular goiter (most likely toxic but radionuclide scanning wasn’t done) with thyrotoxicosis, complicated with thyroid storm, pancytopenia and arrhythmias. All these symptoms resolved after treatment of thyrotoxicosis. Anemia was likely due to the combined effects of both splenic injury and thyrotoxicosis. Decreased WBC and platelet counts were most likely due to thyrotoxicosis as these resolved with treatment of the problem. The bone marrow showed mixed normocellular and few hypercellular fragments. Thus, the bone marrow failure disorders, such as aplastic anemia and myelodysplasia could be ruled out. Drug-induced pancytopenia was also unlikely since patient never took any drug prior to hospital visit. The peripheral blood did not show any typical features of vitamin B12 deficiency such as macrocytosis or neutrophil hypersegmentation. Features of thyroid storm developed on the third day of admission which improved with treatment.

The pathogenesis of pancytopenia in hyperthyroidism is still poorly understood. Immunological mechanisms have been suggested to be involved in reduction of life-span of blood cells and platelets [6]. Two patients were reported by Duquenne et al. who showed signs of macrophage activation with eosinophilia in their bone marrow, which were compatible with an immuno-allergy reaction [6]. Raina et al. described a case of pancytopenia with hypercellular bone marrow related to Graves’ disease [4].

In addition, antineutrophil antibodies and antiplatelet antibodies have been detected in the serum of patients with thyrotoxicosis [7]. Shaw and Mehta reported a case of post-bone marrow transplant pancytopenia which was related to hyperthyroidism [8]. They postulated that thyroid hormone may have a direct effect on hematopoiesis at a stage earlier than erythropoietic stem cell differentiation, disturb maturation and differentiation of the pluripotent stem cells [9]. A reduced marrow granulocyte reserve has also been described in association with hyperthyroidism and attributed to the direct toxicity of the thyroid hormones [9]. Pancytopenia can be a possible manifestation of hyperthyroidism. Although the exact mechanism is still unclear, recovery from hyperthyroidism is associated with resolution of pancytopenia. Thus any patient with unexplained pancytopenia should be investigated to rule out hyperthyroidism.

## Consent

Written informed consent was obtained from the patient for publication of this Case report and any accompanying images. A copy of the written consent is available for review by the Editor of this journal.

## Conclusion

This case describes an important though rare clinical association. Any patient with unexplained pancytopenia should be investigated for thyroid dysfunction. Besides this is the first report which describes this association in a surgical patient.
